# Anticholinergic medication use and falls in postmenopausal women: findings from the women’s health initiative cohort study

**DOI:** 10.1186/s12877-016-0251-0

**Published:** 2016-04-02

**Authors:** Zachary A. Marcum, Heidi S. Wirtz, Mary Pettinger, Andrea Z. LaCroix, Ryan Carnahan, Jane A. Cauley, Jennifer W. Bea, Shelly L. Gray

**Affiliations:** School of Pharmacy, University of Washington, 1959 NE Pacific St, H375G, Box 357630, Seattle, WA 98195-7630 USA; Amgen Inc., One Amgen Center Drive, Thousand Oaks, CA 91320 USA; WHI Clinical Coordinating Center, Fred Hutchinson Cancer Research Center, 1100 Fairview Ave N, Seattle, WA 98109 USA; Division of Epidemiology, UC San Diego, 9500 Gilman Dr #0725, La Jolla, CA 92093 USA; College of Public Health, Department of Epidemiology, University of Iowa, 145 N Riverside Dr, 100 CPHB, Iowa City, IA 52242 USA; Department of Epidemiology, University of Pittsburgh, A510 Crabtree Hall, Pittsburgh, PA 15261 USA; Departments of Medicine and Nutritional Sciences, University of Arizona Cancer Center, 1515 N Campbell Ave, PO Box 245024, Tucson, AZ 85724 USA

**Keywords:** Anticholinergic, Falls, Community dwelling, Older adults

## Abstract

**Background:**

Results from studies assessing the association between anticholinergic use and falls are mixed, and prior studies are limited in their ability to control for important potential confounders. Thus, we sought to examine the association between anticholinergic medication use, including over-the-counter medications, and recurrent falls in community-dwelling older women.

**Methods:**

We analyzed data from a prospective cohort study of women aged 65 to 79 years from the Women’s Health Initiative Observational Study and Clinical Trials. Women were recruited between 1993 and 1998, and analyses included 61,451 women with complete information. Medications with moderate or strong anticholinergic effects were ascertained directly from drug containers during face-to-face interviews. The main outcome measure was recurrent falls (≥2 falls in previous year), which was determined from self-report within 1.5 years subsequent to the medication assessment.

**Results:**

At baseline, 11.3 % were using an anticholinergic medication, of which antihistamines (commonly available over-the-counter) were the most common medication class (received by 45.2 % of individuals on anticholinergic medication). Using multivariable GEE models and controlling for potential confounders, the adjusted odds ratio for anticholinergic medication use was 1.51 (95 % CI, 1.43–1.60) for recurrent falls. Participants using multiple anticholinergic medications had a 100 % increase in likelihood of recurrent falls (adjusted odds ratio 2.00, 95 % CI 1.73–2.32). Results were robust to sensitivity analysis.

**Conclusions:**

Anticholinergic medication use was associated with increased risk for recurrent falls. Our findings reinforce judicious use of anticholinergic medications in older women. Public health efforts should emphasize educating older women regarding the risk of using over-the-counter anticholinergics, such as first-generation antihistamines.

**Electronic supplementary material:**

The online version of this article (doi:10.1186/s12877-016-0251-0) contains supplementary material, which is available to authorized users.

## Background

Falls in older adults are significant public health concerns [1]. Approximately one-third of community-dwelling older adults fall at least once each year, of whom nearly 50 % have recurrent falls [2, 3]. Recurrent falls (as opposed to single falls) may be more clinically important as they may increase the risk of physician visits, functional status decline, nursing home admission, and death [2]. Moreover, the etiology of falls is multifactorial, including both extrinsic (e.g., environmental) and intrinsic (e.g., muscle weakness) risk factors. Importantly, identifying potentially modifiable risk factors for falls is vital in order to reduce the risk of falls in older adults.

Several medication classes, many of which have central nervous system effects, have been associated with increased risk for falls and fractures [4]. Medications with anticholinergic effects have many adverse effects that could contribute to falls risk, including blurred vision, sedation, and cognitive impairment [5, 6]. The prevalence of anticholinergic medication use is common with 13–25 % of community-dwelling older adults taking at least one agent [7–9]. Highly anticholinergic medications are considered as potentially inappropriate for use in older adults [10]. Moreover, nonpharmacological strategies or alternative medications without anticholinergic effects are often available to treat these specific conditions (e.g., sleep disturbances, urinary incontinence); therefore, reducing anticholinergic polypharmacy is feasible.

The prospective cohort studies assessing the association between anticholinergic use and falls in community residing older adults have found mixed results [11–14]. One possible explanation for these mixed results may be due to differences in study designs and in the measurement of falls (e.g., self-reported vs. claims measurement, single vs. recurrent falls). Moreover, anticholinergic use has been reported to be associated with falls in high-risk populations of psychiatric inpatients, patients after traumatic brain injury, and residential care facility residents [15–17]. However, much of the research on anticholinergic use and fall risk among older adults has been limited by use of a cross-sectional design [18] and use of highly select samples that may not be representative to the majority of older adults [15–17]. In addition, most prior research on this topic has been conducted in non-US populations. This is important because in the US over-the-counter medication data are not available in administrative pharmacy claims, resulting in limited prior literature taking this type of medication exposure into account. Thus, additional studies are warranted in large samples to understand potential risks of these medications. The objective of this study was to examine the association between anticholinergic medication use, including over-the-counter medications, and recurrent falls in community-dwelling women.

## Methods

### Study population

The Women’s Health Initiative (WHI) studies included three Clinical Trials (CT) and an Observational Study (OS) that enrolled participants between 1993 and 1998 from 40 clinical centers across the United States. Study methods have been described in detail elsewhere [19]. This analysis included postmenopausal women aged 65–79 years upon study entry; 43,612 women from the Observational Study and 24,427 from the Clinical Trials. We excluded women without a falls assessment within 1.5 years of their baseline medication inventory (*n* = 4033) to ensure the timeliness of the falls relative to medication use, leaving a total sample of 67,006. Informed consent was obtained, and all protocols were approved by the respective institutional review boards at participating institutions.

### Outcome ascertainment: recurrent falls

Fall history was obtained by asking participants to report the number of times they fell and landed on the floor or ground in the past 12 months. Self-reported falls were ascertained annually for women in the OS and every 6 months for women in the CT by use of standardized questionnaires. We defined a participant as having recurrent falls if she reported falling two or more times during the previous 12-month period [20, 21]. This method of fall recall (in the previous 12 months) has been shown to be highly specific (91–95 %) in comparison with that reported using more frequent assessments [22]. All participants (*n* = 67,006) had a baseline falls history collected.

### Anticholinergic medication use

Women were asked about current prescription and over-the-counter medications that were taken in the past 2 weeks at the baseline and year 3 clinic visits. The medication information was obtained directly from the medication containers and entered into the WHI database. Each medication was assigned a drug code using Medispan software (First DataBank, Inc., San Bruno, California). Women were also asked how long they had used each medication. No information on dose was collected.

We adapted the Anticholinergic Drug Scale and focused on medications with moderate to strong anticholinergic activity (see Table 1) [23, 24]. This list was updated by consensus process by study investigators with expertise in pharmacology (co-authors H.W., S.G., R.C.) by using new information (serum anticholinergic activity, receptor binding affinity) and reviewing additional anticholinergic scales [25, 26].

**Table 1:** Frequency of Anticholinergic Medications at Baseline according to Therapeutic Class

Drug Class	Medication^a^	N
Antihistamines		
	Diphenhydramine	5411
	Chlorpheniramine	1408
	Hydroxyzine	742
	Doxylamine	219
	Clemastine	153
	Dexchlorpheniramine	81
	Promethazine	80
	Cyproheptadine	57
Antidepressants		
	Amitriptyline	2585
	Paroxetine	1352
	Nortriptyline	760
	Imipramine	667
	Doxepin	505
	Desipramine	173
Gastrointestinal antispasmodics		
	Anticholinergic combinations	
	Hyoscyamine	1066
	Dicyclomine	516
Urinary antimuscarinics		
	Oxybutynin	983
	Flavoxate	47
Antivertigo/antiemetics		
	Meclizine	924
	Dimenhydrinate	69
	Scopolamine	248
Skeletal Muscle Relaxants		
	Cyclobenzaprine	772
	Orphenadrine	118
Antipsychotics		
	Perphenazine	53
	Trifluoperazine	40
	Thioridazine	38
Antiparkinson agents		
	Benztropine	36
Other		
	Disopyramide	139

^a^Only medications with a frequency of 0.2 % or higher were listed

### Measurement of other covariates

Questionnaires were used to collect information on age, race and ethnicity, history of falls, self-reported health, smoking status, and physical activity from walking outside the home for more than 10 min without stopping (minutes per week). Alcohol consumption was estimated from the responses on the food-frequency questionnaire. Body mass index was calculated from measured height and weight (weight, kg/height, m^2^). Physical function was measured from the 10-item Rand Physical Function scale (>90 indicating higher function) [27].

Anticholinergics can be used for a variety of conditions. Therefore, to control for potential confounding by indication we measured self-reported of physician-diagnosed medical conditions for which anticholinergics may be used – urinary incontinence and Parkinson’s disease. Other medical conditions measured to address potential confounding by indication included insomnia and depressive symptoms. The 5-item WHI Insomnia Rating Scale was used for perceived insomnia symptoms, including sleep latency, sleep maintenance insomnia, early morning awakening, and sleep quality [28]. For each question, the score ranges from 0 to 4, and the summary score ranges from 0 to 20, with higher scores indicate greater insomnia. We classified the score for insomnia into four categories (0–3, 4–6, 7–10, and ≥11) consistent with a previous study [28]. The Centers for Epidemiologic Studies-Depression 6-item questionnaire was used to assess depressive symptoms (Burnham score >0.06) [29, 30].

In addition, diabetes was defined as presence of oral hypoglycemic medication or insulin, and Alzheimer Disease was measured via self-report of physician-diagnosed disease and/or use of an anti-dementia medication (i.e., acetylcholinesterase inhibitors or NMDA receptor antagonist). Arthritis was measured via self-report of a physician diagnosis. Baseline medication covariates included non-anticholinergic antiepileptics and psychoactive medications (benzodiazepine receptor agonists, antidepressants, and antipsychotics). Self-reported moderate/severe dizziness, back pain (moderate severe), and uncorrected vision problems were also assessed.

### Statistical analysis

We used appropriate descriptive statistics for summarization and generalized estimating equations (GEE) for eliciting the main findings, allowing for multiple observations per participant. Odds ratios and 95 % confidence intervals were estimated from the GEE models that included baseline and year 3 medication exposure information, and recurrent falls in the following year as the outcome. Covariate data were from baseline. We included only women without missing covariate data for the primary analysis (*N* = 61,451). The primary analysis examined any use of anticholinergics as the main independent variable of interest and the risk of recurrent falls reported in 1.5 years subsequent to the assessment as the outcome. Since the focus of the analysis was on falls most proximal to medication use, only the most recent fall assessment (within 1.5 years) was used. Baseline and year 3 anticholinergic use was used to define the exposure in the primary analyses. All models were adjusted by age using 5-year age intervals and study component (clinical trial vs. observational study). We examined three models: Model 1 was adjusted for linear age at screening, diet modification (DM) trial, and Calcium/vitamin D (CaD) trial randomization arms; Model 2 was adjusted as Model 1, with additional adjustment for race/ethnicity, body mass index, poor vision, arthritis, treated diabetes, low back pain, Alzheimer Disease and/or use of anti-dementia medication, Parkinson’s Disease, urinary incontinence, depression, insomnia, self-reported health, alcohol intake, use of antiepileptics, and number of psychoactive medications. Most prior studies have been limited in their ability to adjust for physical function or physical activity, which are potential confounders. Because of a prevalent user design, these factors could also be influenced by anticholinergic use (or in the causal pathway). In addition, dizziness could be a confounder or a side effect from anticholinergic use. To examine this issue, we assessed a separate model (Model 3) that included all variables from Model 2, with additional adjustment for physical function, current physical activity from walking, and dizziness.

In addition, we assessed the association between the number of concurrent anticholinergic medications (0, 1 or ≥2), and duration of anticholinergic use at baseline (<1 year, 1–3 years, >3 years) and the risk of recurrent falls. We conducted trend tests for the number and duration of anticholinergic medication variables in order to assess associations across categories. Given the heterogeneity in medication use patterns (i.e., chronic vs. intermittent use as seen with many antihistamines) across anticholinergic agents, we grouped anticholinergic sub-classes into similar therapeutic groups (i.e., antihistamines, antidepressants/antipsychotics, gastrointestinal antispasmodics, urinary antimuscarinics, antivertigo/antiemetic, and miscellaneous) and assessed the association between each group and recurrent falls controlling for use of anticholinergics in other sub-classes. In addition to the primary associations of interest, we developed an interaction model to evaluate effect modification for recurrent falls based on history of a fall.

A sensitivity analysis was conducted to evaluate the robustness of our study results. Because the clinical trial arms of the WHI did not update many of the covariates at year 3, we ran the primary analyses only on those participants in the observational study sample, and updated the covariates at the year 3 exposure. All analyses were conducted using SAS statistical software, version 9.2 (SAS Institute Inc, Cary, North Carolina) with the GENMOD procedure to run the GEE models.

## Results

Among the baseline sample of 61,451 postmenopausal women with complete data, the majority was white and overweight or obese (Table 2). At baseline, 11.3 % older women were using an anticholinergic medication (Table 3). Of users at baseline, 25.6 % had reported using the medication for less than 1 year, 36.2 % had used from 1 to 3 years, and 38.2 % had used for more than 3 years. Use of multiple anticholinergic medications was reported by 8.8 % of users. Compared to nonusers, individuals on anticholinergic medication were more likely to be obese, have a history of falls, have several health conditions (e.g., urinary incontinence, arthritis, low back pain), have higher psychoactive medication use including use certain medications (e.g., benzodiazepines, antidepressants), and have poorer physical function and poor/fair self-reported health.

**Table 2:** Baseline Characteristics of Sample: Overall and by Anticholinergic Use^a^ (*n* = 61,451)

	Overall		User of AC		Non-use of AC	
	(*n* = 61,451)		(*n* = 6940)		(*n* = 54,511)	
	N	%	N	%	N	%
Age group at screening						
65–69	31,008	50.5	3427	49.4	27,581	50.6
70–74	21,491	35.0	2486	35.8	19,005	34.9
75+	8952	14.6	1027	14.8	7925	14.5
Race/ethnicity						
White	54,015	87.9	6264	90.3	47,751	87.6
Black	3572	5.8	314	4.5	3258	6.0
Hispanic	1241	2.0	160	2.3	1081	2.0
American Indian	200	0.3	34	0.5	166	0.3
Asian/Pacific Islander	1594	2.6	82	1.2	1512	2.8
Unknown	829	1.4	86	1.2	743	1.4
Number of falls in last 12 months (missing = 1658)						
None	40,419	67.6	4194	61.8	36,225	68.3
1	12,200	20.4	1442	21.3	10,758	20.3
2	4946	8.3	734	10.8	4212	8.0
3 or more	2228	3.7	415	6.1	1813	3.4
Fair or poor self-reported health	5426	8.8	1049	15.1	4377	8.0
Smoking status (missing = 698)						
Never	32,298	53.2	3486	51.0	28,812	53.4
Past	25,459	41.9	3017	44.1	22,442	41.6
Current	2996	4.9	337	4.9	2659	4.9
Minutes per week spent walking (missing = 137)						
0 min	19,045	31.1	2494	36.0	16,551	30.4
>0–150 min	32,225	52.6	3529	51.0	28,696	52.8
>150 min	10,044	16.4	903	13.0	9141	16.8
Alcohol intake						
Non drinker	7375	12.0	852	12.3	6523	12.0
Past drinker	11,657	19.0	1615	23.3	10,042	18.4
<7 drinks per week	34,646	56.4	3665	52.8	30,981	56.8
7+ drinks per week	7773	12.7	808	11.6	6965	12.8
BMI (kg/m^2^)						
Underweight (<18.5)	619	1.0	58	0.8	561	1.0
Normal (18.5–24.9)	21,919	35.7	2184	31.5	19,735	36.2
Overweight (25.0–29.9)	22,341	36.4	2601	37.5	19,740	36.2
Obese (≥30.0)	16,572	27.0	2097	30.2	14,475	26.6
Rand Physical Function score >90 (missing = 731)	16,870	27.8	1191	17.4	15,679	29.1
Urinary incontinence	19,244	31.3	2754	39.7	16,490	30.3
History of Parkinson’s disease	228	0.4	64	0.9	164	0.3
Insomnia Rating Scale						
0–3	15,317	24.9	1296	18.7	14,021	25.7
4–6	17,171	27.9	1698	24.5	15,473	28.4
7–10	16,493	26.8	2034	29.3	14,459	26.5
≥11	12,470	20.3	1912	27.6	10,558	19.4
Depression (Burnham score >0.06)	5230	8.5	941	13.6	4289	7.9
Treated diabetes (oral or insulin)	2962	4.8	454	6.5	2508	4.6
Alzheimer Disease	35	0.1	2	0.03	33	0.1
Arthritis	34,728	56.5	4746	68.4	29,982	55.0
Anti-epileptic use	605	1.0	152	2.2	453	0.8
Benzodiazepine receptor agonist use	2563	4.2	860	12.4	1703	3.1
Antidepressant use	1710	2.8	409	5.9	1301	2.4
Antipsychotic use	54	0.1	27	0.4	27	0.1
Number of psychoactive meds						
0	57,378	93.4	5723	82.5	51,655	94.8
1	3666	6.0	1072	15.5	2594	4.8
2+	407	0.7	145	2.1	262	0.5
Dizziness (moderate/severe) (missing = 156)	1829	3.0	412	6.0	1417	2.6
Low back pain (moderate/severe)	12,719	20.7	2160	31.1	10,559	19.4
Vision problem uncorrected (moderate/severe)	3560	5.8	550	7.9	3010	5.5
WHI Participation						
Hormone therapy trial	10,986	17.9	1180	17.0	9806	18.0
Diet modification trial	16,497	26.9	1754	25.3	14,743	27.1
Calcium vitamin D trial	12,706	20.7	1272	18.3	11,434	21.0
Observational study	36,723	59.8	4288	61.8	32,435	59.5

Abbreviation: *AC* anticholinergic

^a^Missing value is 0 unless otherwise specified

**Table 3:** Descriptives of Anticholinergic Medication Use Prevalence at Baseline and Year 3

	Baseline		Year 3	
	N	%	N	%
Any Use	6940	100.00	6985	100.00
Number of Agents				
1	6332	91.24	6333	90.67
2+	608	8.76	652	9.33
Duration				
<1 year	1775	25.58	1786	25.57
1–3 years	2514	36.22	3306	47.33
>3 years	2651	38.20	1893	27.10
Specific Anticholinergic Class				
Antihistamines	3136	45.19	2355	33.72
Antidepressants/Antipsychotics	2252	32.45	2381	34.09
Gastrointestinal antispasmodics	670	9.65	499	7.14
Urinary antimuscarinics	582	8.39	1653	23.66
Antivertigo/antiemetics	521	7.51	429	6.14
Miscellaneous^a^	344	4.96	301	4.31

^a^Miscellaneous: antiparkinson agents and disopyramide

Using multivariable GEE models and controlling for potential confounders, the adjusted odds ratio for any anticholinergic medication use was 1.51 (95 % CI, 1.43–1.60) for recurrent falls (Table 4). Adjustment for physical function, physical activity, and dizziness had negligible impact on the odds ratio (Model 2 versus Model 3, Table 4). Participants using ≥ 2 anticholinergic medications had a 100 % increase in likelihood of recurrent falls (adjusted odds ratio [AOR] 2.00, 95 % CI 1.73–2.32). A significant trend for increasing likelihood of recurrent falls was detected with increasing duration of anticholinergic medication use (trend test *p*-value <0.0001). Moreover, use of each anticholinergic sub-class was associated with a statistically significant increase in likelihood of recurrent falls, with antidepressants/antipsychotics having the largest adjusted odds ratio (AOR 1.81, 95 % CI, 1.66–1.97) among the sub-classes.

**Table 4:** Adjusted Odds Ratios Relating Anticholinergic Use to Incident Recurrent Falls over the next 12 months^a^

		Model 1		Model 2		Model 3
	Number With falls	OR (95 % CI)	Number With falls	OR (95 % CI)	Number With falls	OR (95 % CI)
Any Anticholinergic use						
No use	7883	1.0	7883	1.0	7743	1.0
Any use	1938	1.86 (1.76, 1.97)	1938	1.53 (1.45, 1.62)	1903	1.51 (1.43, 1.60)
Number of Agents^*^						
No use	7883	1.0	7883	1.0	7743	1.0
1	1688	1.78 (1.67, 1.88)	1688	1.48 (1.40, 1.57)	1657	1.47 (1.38, 1.56)
2+	250	2.84 (2.45, 3.28)	250	2.04 (1.76, 2.36)	246	2.00 (1.73, 2.32)
Duration^*^						
No use	7883	1.0	7883	1.0	7743	1.0
<1 year	495	1.82 (1.64, 2.01)	495	1.52 (1.37, 1.68)	485	1.50 (1.36, 1.66)
1–3 years	760	1.73 (1.59, 1.88)	760	1.42 (1.31, 1.55)	745	1.41 (1.29, 1.53)
>3 years	683	2.10 (1.92, 2.29)	683	1.70 (1.55, 1.86)	673	1.68 (1.53, 1.84)
Specific Anticholinergic Class^b^						
Antihistamine/Antiemetic/Antivertigo	742	1.39 (1.28, 1.51)	742	1.22 (1.12, 1.32)	734	1.21 (1.11, 1.32)
Antidepressant/Antipsychotic	811	2.26 (2.07, 2.46)	811	1.83 (1.67, 1.99)	796	1.81 (1.66, 1.97)
Gastrointestinal/Urinary Antimuscarinics/Miscellaneous	607	1.81 (1.64, 1.99)	607	1.49 (1.35, 1.64)	591	1.47 (1.33, 1.62)

Abbreviations: *CI* confidence intervals, *OR* odds ratio

^*^Trend test *p*-values all <0.0001

^a^OR and CI are estimated from GEE models using an unstructured correlation matrix, including baseline and year 3 medication exposure information, and recurrent falls in the following year. All models are adjusted by age using 5-year age intervals, and study component (clinical trial vs. observational study). Model 1 adjusted for linear age, diet modification and CaD trial randomization arms. Model 2 was further adjusted for race/ethnicity, body mass index, poor vision, arthritis, treated diabetes, low back pain, Alzheimer’s Disease diagnosis and/or use of medication, indications for use (Parkinson’s disease, urinary incontinence, depression, insomnia scale), self-reported health, alcohol intake, use of antiepileptics, and number of psychoactive medications. Model 3 was further adjusted for physical function, dizziness, current physical activity from walking. All adjustment variables are from baseline

^b^Odds ratios for type of anticholinergic medication are adjusted for simultaneous use of a different type

Our sensitivity analysis did not reveal a statistically significant effect modification for recurrent falls based on history of a fall (falls in prior year: none/<2, AOR 1.47, 95 % CI, 1.37–1.58; falls in prior year: ≥2, AOR 1.38, 95 % CI, 1.25–1.53; interaction p value, 0.32). In addition, the sensitivity analysis including updated covariates at year 3 revealed similar findings to the primary analysis.

## Discussion

We found that anticholinergic medication use was associated with recurrent falls among older postmenopausal women. The association with recurrent falls was even greater among those using multiple anticholinergic medications. In addition, we found a significant association between longer duration of anticholinergic use and recurrent falls. Each of the anticholinergic sub-classes was associated with recurrent falls; thus even anticholinergics likely to be used intermittently (e.g. antihistamines) were associated with recurrent falls. These findings were robust to sensitivity analysis.

A number of authoritative geriatric medicine sources report that anticholinergic use may increase fall risk [10, 31], despite some inconsistencies in the data that support these recommendations. Thus, our study addresses an important gap in the literature by evaluating the association between anticholinergic medication use and risk for falls among a large sample of community-dwelling women. The most comparable study to the current one was conducted in 2948 community-dwelling older men and women as part of the Health, Aging and Body Composition (ABC) study [13]. These authors also found an increased risk of recurrent falls in individuals on anticholinergic medication, but the results were not statistically significant perhaps because of the smaller sample size.

Conversely, a study by Berdot et al. on 6343 community-dwelling men and women aged ≥65 years found that the risk of falls was not significantly increased among individuals on anticholinergic medication in adjusted analysis [11]. However, when stratifying the analysis by type of anticholinergic use, Berdot et al. reported a statistically significant increase in likelihood of falls for regular but not occasional users compared to non users [11]. Of note, the authors were not able to determine if anticholinergic use had occurred before or after the fall based on their operational definition of regular use. Richardson et al. assessed the association between anticholinergic use and self-reported injurious falls in community-dwelling men and women aged ≥65 years in Ireland and found a statistically significant association between regular use and subsequent self-reported injurious falls in men, but not women [14]. In addition, Nisthala et al. examined the impact of anticholinergic and sedative medications (as measured by the Drug Burden Index [DBI]) in people aged ≥65 years in New Zealand and found that exposure to DBI drugs was independently associated with fall-related hospitalizations, primary care visits, and mortality [12]. However, it was not possible to disentangle the independent effect of anticholinergic use from the overall DBI drug exposure. While the literature regarding anticholinergic use and falls in older adults remains somewhat mixed, mounting evidence – including the results from our current study – suggests a significant association.

Given that many anticholinergic medications are over-the-counter, they may be used only intermittently (e.g., antihistamines). Medications with anticholinergic properties may increase falls risk by several mechanisms, including blurred vision, sedation, and cognitive impairment, therefore it is plausible that even intermittent use could increase risk via these adverse events [5, 6]. We explored this issue by performing our sub-class analysis where we found that even antihistamines were significantly associated with falls. It is also important to note that since antihistamines were the most commonly used therapeutic class of anticholinergics, studies relying on claims data to measure medication exposure would not capture antihistamines purchased over-the-counter and used intermittently. Future research would be strengthened by examining the association between patterns of anticholinergic use (including sporadic use) and falls.

Strengths of this study include the large sample of diverse women and extensive covariate information. In particular, we were able to adjust for important potential confounders not available in most administrative claims datasets, including physical function, physical activity, and dizziness. However, there are several limitations that warrant discussion. First, multiple scales exist for measuring anticholinergic exposure, with no true gold standard [32]. A study by Naples et al. found only low to moderate concordance among five commonly used anticholinergic scales [33]. To address this potential limitation, we included medications with moderate and strong anticholinergic activity and avoided medications with more questionable anticholinergic activity, potentially leading to a lower prevalence of anticholinergic medication use and thus a conservative estimate of the association between anticholinergic use and recurrent falls. Furthermore, our list is very similar to the medications identified as highly anticholinergic on the 2012 updated Beers criteria [10]. Medication information was only collected at baseline and year 3. As a result, anticholinergics users were likely to be those who tolerated the early adverse effects, and women experiencing adverse events may have discontinued the anticholinergics prior to their capture in our periodic assessment of medication use. This would be expected to bias our results towards the null and, therefore, our results are likely conservative. Finally, we did our best to adjust for baseline differences, but like all observational studies, residual or unmeasured confounding could explain our findings.

## Conclusions

In conclusion, use of medications with strong or moderate anticholinergic effects in older women is associated with a slightly higher risk of recurrent falls, especially in those taking multiple agents or for 3 or more years. Anticholinergic medications have several potential adverse events (e.g., memory impairment, constipation, urinary retention), yet they continue to be widely used. Health care providers are encouraged to use anticholinergic medications judiciously and resort to alternative therapies when available. Since many medications with anticholinergic effects are available without a prescription, an effort to increase awareness among older adults of the potential for increased falling risk is an important public health priority.

### Ethics, consent and permissions

All procedures performed in studies were approved by the respective institutional review boards at participating institutions and were in accordance with the 1964 Declaration of Helsinki and its later amendments or comparable ethical standards. Specifically, the study protocol for the Women’s Health Initiative was reviewed and approved by each clinical center’s institutional review board (see Additional file 1). Informed consent was obtained from all individual participants included in the study.

### Availability of data and materials

The datasets supporting the conclusions of this article are available at: https://www.whi.org/SitePages/WHI%20Home.aspx.

## Additional file

Additional file 1: WHI Clinical Centers (DOCX 111 kb)

## Abbreviations

ABC: aging, and body composition
ADS: anticholinergic drug scale
AOR: adjusted odds ration
CaD: calicum/vitamin D
CT: clinical trials
DBI: drug burden index
DM: diet modification
GEE: generalized estimating equations
OS: observational study
WHI: women’s health initiative
