# Supplementary material for: Anticholinergic medication use and falls in postmenopausal women: findings from the women’s health initiative cohort study
Source: BMC Geriatr. 2016 Apr 2;16:76. doi: 10.1186/s12877-016-0251-0 (PMC4818856; doi:10.1186/s12877-016-0251-0)
Supplement: Additional file 1: — WHI Clinical Centers (DOCX 111 kb) [file 12877_2016_251_MOESM1_ESM.docx]

**WHI Clinical Centers**

| 1) University of Alabama at Birmingham  2) University of Arizona  3) Kaiser Permanente California  4) Harbor-UCLA Research and Education Institute  5) University of California, Irvine  6) UCLA Medical Center  7) LaJolla Vanguard Clinical Center  8) General Internal Medicine Investigative Clinic, University of California, Davis  9) Stanford University  10) University of Florida  11) University of Miami  12) Atlanta Vanguard Clinical Center  13) Hawaii Center for Health Research  14) Chicago-Westside Clinical Center Northwestern University  15) Northwestern University  16) University of Iowa  17) Brigham and Women's Hospital  18) University of Massachusetts Medical School  19) Wayne State University  20) Berman Center for Clinical Research  21) University of Nevada School of Medicine  22) University of Medicine and Dentistry of N.J.  23) State University of NY at Stony Brook  24) Albert Einstein College of Medicine  25) State University of NY at Buffalo  26) University of North Carolina at Chapel Hill  27) Department of Public Health Sciences, Section on Epidemiology, Wake Forest University  28) University of Cincinnati Medical Center  29) Ohio State University  30) Kaiser Permanente Oregon  31) University of Pittsburgh  32) The Memorial Hospital of Rhode Island  33) University of Tennessee, Memphis  34) University of Texas Health Science Center at San Antonio  35) Baylor College of Medicine  36) MedStar Research Institute  37) Lipid Research Clinic George Washington University  38) Fred Hutchinson Cancer Research Center  39) Medical College of Wisconsin  40) University of WI, Madison |
| --- |
